# RioCC: Efficient and Accurate Class-Level Code Recommendation Based on Deep Code Clone Detection

**DOI:** 10.3390/e28020223

**Published:** 2026-02-14

**Authors:** Hongcan Gao, Chenkai Guo, Hui Yang

**Affiliations:** 1 School of Information Engineering, Tianjin University of Commerce, Tianjin 300133, China; 2 Anhui Province Key Laboratory of Cyberspace Security Situation Awareness and Evaluation, College of Cryptology and Cyber Science, Nankai University, Tianjin 300350, China; guochenkai@nankai.edu.cn; 3 360 Intelligence (Zhuhai Hengqin) Technology Co., Ltd., Zhuhai 519000, China; yanghui1@360.cn

**Keywords:** code recommendation, class-level code, deep forest, code clone detection, coarse-to-fine candidate reduction

## Abstract

**Context:** Code recommendation plays an important role in improving programming efficiency and software quality. Existing approaches mainly focus on method- or API-level recommendations, which limits their effectiveness to local code contexts. From a multi-stage recommendation perspective, class-level code recommendation aims to efficiently narrow a large candidate code space while preserving essential structural information. **Objective:** This paper proposes RioCC, a class-level code recommendation framework that leverages deep forest-based code clone detection to progressively reduce the candidate space and improve recommendation efficiency in large-scale code spaces. **Method:** RioCC models the recommendation process as a coarse-to-fine candidate reduction procedure. In the coarse-grained stage, a quick search-based filtering module performs rapid candidate screening and initial similarity estimation, effectively pruning irrelevant candidates and narrowing the search space. In the fine-grained stage, a deep forest-based analysis with cascade learning and multi-grained scanning captures context- and structure-aware representations of class-level code fragments, enabling accurate similarity assessment and recommendation. This two-stage design explicitly separates coarse candidate filtering from detailed semantic matching to balance efficiency and accuracy. **Results:** Experiments on a large-scale dataset containing 192,000 clone pairs from BigCloneBench and a collected code pool show that RioCC consistently outperforms state-of-the-art methods, including CCLearner, Oreo, and RSharer, across four types of code clones, while significantly accelerating the recommendation process with comparable detection accuracy. **Conclusions:** By explicitly formulating class-level code recommendation as a staged retrieval and refinement problem, RioCC provides an efficient and scalable solution for large-scale code recommendation and demonstrates the practical value of integrating lightweight filtering with deep forest-based learning.

## 1. Introduction

Software development often involves reusing existing code fragments to improve efficiency and maintainability [1]. A well-designed code recommendation tool can significantly streamline the development process by retrieving relevant code snippets from a source code pool and identifying potential coding mistakes [2]. Such tools contribute to improving software quality and accelerating development by providing programmers with useful code references.

Various approaches have been proposed for code recommendation, including code-to-code search tools [2,3,4], pattern-based code completion [5,6,7], model-driven engineering [8], and clone detection [9]. Code-to-code search tools retrieve relevant snippets based on input queries, while pattern-based approaches mine recurring patterns from large code corpora to suggest relevant extensions. Model-driven engineering techniques leverage abstract models and domain-specific languages to generate or recommend code artifacts. Recently, clone-based recommendation methods have gained attention [10,11] due to their ability to identify structurally and semantically similar code fragments.

Unlike code-to-code search and pattern-based recommendation, clone detection focuses on locating similar code fragments within a given code pool. Advanced clone detection techniques can improve recommendation accuracy by capturing structural and semantic similarities beyond simple text matching [12,13]. In our work, we deliberately adopt a class-level scope as a design choice. While most existing efforts primarily focus on method- and API-level recommendations [14,15,16], class-level recommendations encapsulate complete code structures and provide a broader semantic context. This design enables developers to better understand, reuse, and maintain related implementations, particularly when working with complex software systems. However, despite these advantages, existing clone-based recommendation methods suffer from several limitations: **(1) Query Extension Challenge**: Clone-based recommendations often retrieve code snippets that are nearly identical to the query but lack additional useful extensions [2]. This limits their utility for recommending complete and functionally relevant code. **(2) Detection of Complex Clones**: While clone detection techniques excel at identifying textual (Type-1) and lexical (Type-2) clones, they struggle with syntactic (Type-3) and semantic (Type-4) clones due to structural complexity [17]. **(3) Efficiency Concerns**: The computational overhead of clone detection is often overlooked, making existing approaches impractical for large-scale code repositories. Our investigation (Section 4.3) reveals that traditional clone-based recommendation models [10,18,19] suffer from unacceptable time consumption when applied to large code pools.

To address these challenges, we propose **RioCC** (Recommending sImilar cOdes via Code Clones), a novel class-level code recommendation approach that leverages deep learning techniques for efficient and accurate code retrieval. RioCC operates in three main stages: **(1) Quick Search**: The source code is transformed into abstract syntax tree (AST) representations and encoded for fast similarity computation. **(2) Clone Detection:** A deep forest model is trained to identify four types of code clones at the class level. **(3) Code Recommendation:** The most relevant class-level code snippets are ranked and provided to developers based on computed similarities. Extensive experiments on BigCloneBench and real-world code fragments demonstrate that RioCC achieves state-of-the-art performance in both recommendation accuracy and efficiency.

Our contributions can be summarized as follows:

- We introduce a class-level code recommendation framework that bridges the gap between clone detection and practical recommendation. Unlike method-level approaches, it retrieves relevant yet structurally diverse code snippets, effectively enabling query expansion and providing developers with a broader and more useful set of recommendations.
- We employ a deep forest model to enhance representation learning for clone detection. This model captures both context and structure, improving the detection of complex (Type-3 and Type-4) clones.
- We integrate a quick search module based on matrix computations to efficiently filter out irrelevant candidates, significantly reducing the time complexity of the recommendation process.

The rest of this paper is organized as follows: Section 2 details the RioCC framework. Section 3 describes the datasets and experimental setup. Section 4 presents the experimental results and the analysis of the research question. Section 5 discusses potential limitations. Section 6 discusses the comparison of RioCC with LLM-based approaches for code recommendation. Section 7 reviews related studies. Finally, Section 8 concludes our work.

## 2. Approach

### 2.1. Overview

**Figure 1:**
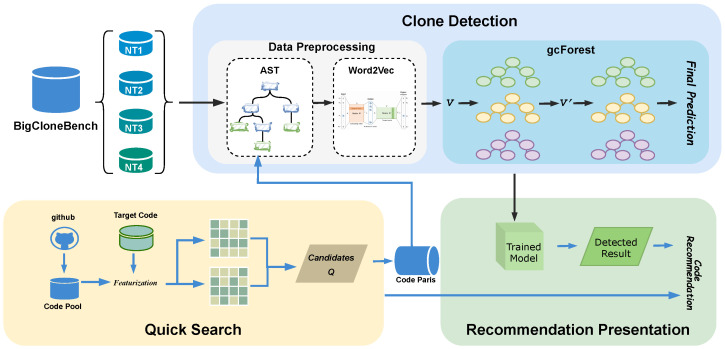
Overview of the RioCC framework, which includes Quick Search, Clone Detection, and Recommendation Presentation.

Figure 1 illustrates the overall architecture of RioCC, which consists of three key stages: (1) **Quick Search**, (2) **Clone Detection**, and (3) **Recommendation Presentation**. The framework is designed to efficiently retrieve and recommend class-level code fragments that exhibit high structural and semantic similarity to a given query code fragment.

Given a target code fragment $C^{t}$, the objective of the first stage is to efficiently retrieve a set of top-$N_{\#}$ candidate code fragments from a large code repository $C^{P}$ that exhibit high structural overlap with $C^{t}$. To achieve **Quick Search**, we first transform all code fragments in $C^{P}$ into their corresponding AST representations. Next, we apply one-hot encoding to the AST nodes, resulting in a sparse feature matrix $F_{C^{P}}^{*}$ for the code pool and a feature vector $F_{C^{t}}^{*}$ for the query code. The quick search similarity score, denoted as $SQ_{\mathrm{score}}$, is then computed via a matrix multiplication between $F_{C^{t}}^{*}$ and $F_{C^{P}}^{*}$, enabling efficient ranking of candidate code fragments. The top-$N_{\#}$ candidates are selected for further analysis.

In the **Clone Detection** stage, we leverage a deep forest model to enhance clone identification accuracy. We first collect a large-scale dataset of labeled code clone pairs from BigCloneBench. Since raw code fragments cannot be directly processed for model training, we transform them into AST representations to retain both syntactic and semantic information. The AST nodes are then embedded using Word2Vec, producing dense vector representations *V*. Word2Vec is adopted for its low computational cost, scalability to large datasets, and straightforward integration with AST-based representations, which facilitates efficient processing in large-scale settings. In contrast, models such as Code2Vec [20] rely on path-based representations and involve more complex preprocessing pipelines, increasing implementation complexity in large-scale scenarios. Recent pretrained code models, such as CodeBERT [21] or GraphCodeBERT [22], represent alternative embedding backbones with strong contextual modeling capabilities, but their integration and evaluation are beyond the scope of this work and are left for future exploration.

These representations are then fed into a pre-trained *gcForest* model for classification. The model architecture consists of a multi-grained scanning module, which refines the vector representations into $V^{\prime }$, followed by a cascade forest module that classifies code clones into four types (Type-1 to Type-4). Based on the classification results, a similarity score $SC_{\mathrm{score}}$ is assigned to each candidate code fragment. Representing ASTs in a linearized form may lead to the loss of certain hierarchical or relational structural information, which could be more naturally captured by tree- or graph-based encoders.

Finally, in the **Recommendation Presentation** stage, a statistical aggregation function combines the quick search similarity score $SQ_{\mathrm{score}}$ and the clone detection similarity score $SC_{\mathrm{score}}$ to compute a final ranking of candidate code fragments. The top-*N* ranked fragments are then recommended to the programmer, providing relevant and structurally diverse code suggestions.

The following sections provide a detailed description of each module in the RioCC framework.

### 2.2. Quick Search

**Figure 2:**
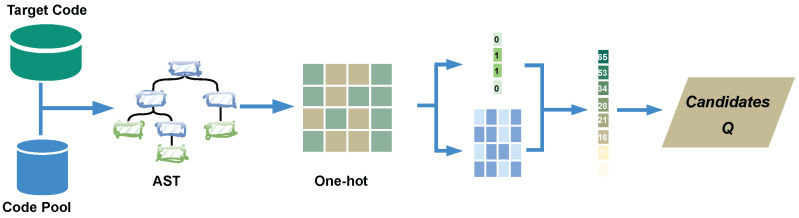
Overview of the Quick Search Process.

The goal of the Quick Search stage is to efficiently retrieve the top-$N_{\#}$ candidate code fragments from a large code corpus that exhibit structural similarity to a given target code fragment $C^{t}$. This is achieved by computing an initial similarity score between $C^{t}$ and all code fragments in the repository $C^{P}$, as shown in Figure 2.

To this end, RioCC first converts all code fragments in $C^{P}$ into their corresponding AST representations. The AST serves as an abstract representation of the syntactic structure of a program, where each node in the tree corresponds to a meaningful token, including lexical markers, syntactic constructs, and semantic information. Let $F_{C^{P}}$ denote the set of features extracted from all code fragments in $C^{P}$, and let $F_{C^{t}}$ represent the features extracted from the target code fragment.

For each candidate code fragment $C^{Pi}\in C^{P}$, the initial similarity between $C^{t}$ and $C^{Pi}$ is computed as the intersection of their feature sets:

(1) $$S\left(F_{C^{t}}\right)\cap S\left(F_{C^{Pi}}\right),$$

where S(·) denotes the set of extracted features. The top-$N_{\#}$ candidate code fragments are selected based on this similarity score.

To facilitate efficient similarity computation, we encode the extracted AST tokens as feature vectors. Each AST token is initially encoded using a one-hot encoding scheme, resulting in a binary feature representation of length |L|. Consequently, the feature vectors of all code fragments in $C^{P}$ can be represented as a matrix $F_{C^{P}}^{*}$ of dimension $|F_{C^{P}}^{*}|\times |L|$, while the target fragment $C^{t}$ is encoded as $F_{C^{t}}^{*}$.

The similarity score for each candidate code fragment is then computed via matrix multiplication:

(2) $$F_{C^{P}}^{*}\cdot F_{C^{t}}^{*},$$

which results in a vector of size $|F_{C^{P}}^{*}|$, where each entry represents the similarity between $C^{t}$ and a code fragment in $C^{P}$. The top-$N_{\#}$ candidates are then selected via a simple sorting operation, forming the set *Q*.

Given that the code repository contains over a million features, with each code fragment typically consisting of fewer than 100 features, the resulting feature matrix is highly sparse. Consequently, the matrix multiplication operation in Equation (2) can be computed efficiently in a short time. Moreover, $F_{C^{P}}^{*}$ can be precomputed offline, further enhancing the efficiency of retrieving the top-$N_{\#}$ candidates and reducing the computational burden of the subsequent clone detection stage.

### 2.3. Clone Detection

**Figure 3:**
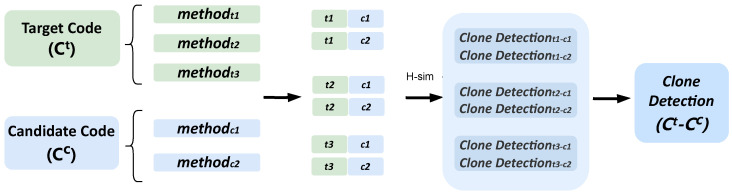
Illustration of class-level clone detection via method-level matching. Clone types between a target class and a candidate class are determined by aggregating method-level clone detection results, where the highest-similarity method pairs are used to infer the overall class-level clone type.

The objective of this step is to identify highly similar code fragments within a given code repository using an accurate clone detection model. This allows us to compute fine-grained code similarity—referred to as code clone-based similarity—between the candidate list *Q* and the target file $C^{t}$. The overall architecture of our clone detection module is illustrated in Figure 3.

Due to the significant variability in class file sizes, we adopt a bottom-up approach for class-level clone detection. Specifically, we first compute clone detection results for method pairs within the involved classes and subsequently determine the clone type of the target class based on these pairwise results.

Formally, we extract all methods from the target code fragment $C^{t}$ and from each candidate code fragment in *Q*. Let $C^{t}$ contain three methods, denoted as $method_{t1}$, $method_{t2}$, and $method_{t3}$, and let a candidate fragment $C^{c}\in Q$ contain two methods, denoted as $method_{c1}$ and $method_{c2}$. By considering all possible method pairs across $C^{t}$ and $C^{c}$, we obtain six clone detection results: *clone detection*_*t*1−*c*1_, *clone detection*_*t*1−*c*2_, *clone detection*_*t*2−*c*1_, *clone detection*_*t*2−*c*2_, *clone detection*_*t*3−*c*1_, and *clone detection*_*t*3−*c*2_.

For each method $t_{i}$ in $C^{t}$, we select the highest similarity score among its detection results with all methods in $C^{c}$, denoted as *H-sim* in Figure 3. Finally, we determine the clone type of $C^{t}$ and $C^{c}$ based on the most frequently occurring clone type across these highest-similarity method pairs.

Following prior works on code clone classification [10,18], we categorize code clones into four distinct types:

- **Type-1 (Textual Similarity)**: Two code fragments are identical except for differences in spaces, comments, and layout. This type is also referred to as an *“exact clone”*.
- **Type-2 (Lexical or Token-Based Similarity)**: These clones differ in identifier names, variable names, type names, and function names but retain the same structure. This category is also known as a *“renamed/parameterized clone”*.
- **Type-3 (Syntactic Similarity)**: Code fragments exhibit insertions or deletions of statements while still maintaining similar syntactic structures. Additionally, differences may exist in identifiers, types, spaces, layout, and comments. This type is also referred to as a *“near-miss clone”* or *“gapped clone”*.
- **Type-4 (Semantic Similarity)**: Two code fragments are syntactically dissimilar but functionally equivalent. This type is also known as a *“semantic clone”*.

We formulate the code clone detection task as a multi-class classification problem. To achieve this, we integrate a deep forest model into our detection framework, comprising data preprocessing and the construction of a gcForest-based classification model.

#### 2.3.1. Data Preprocessing

Since raw code fragments cannot be directly utilized for model training, we first perform a data preprocessing step, which consists of two key phases: AST extraction and Word2Vec transformation.

To this end, we collect a large number of labeled code clone pairs from BigCloneBench [23] and convert the code into AST representations, preserving both semantic and syntactic information. The ASTs are then serialized using a preorder traversal to convert the tree structures into linear sequences of node types, which are subsequently encoded as one-hot vectors for further processing.

Subsequently, we employ Word2Vec [24], a widely used unsupervised learning technique, to transform the structured AST data into low-dimensional numerical vector representations while retaining the essential semantic and syntactic information. In particular, we utilize the skip-gram model [25] for this transformation. Given a sequence of words $w_{1},w_{2},w_{3},\ldots ,w_{n}$, the objective of the skip-gram model is to maximize the following likelihood function:

(3) $$L\left(t\right)=\frac{1}{T}\sum_{t=1}^{T}\sum_{-c\leq i\leq c,i\neq 0}\log p\left(w_{t+i}|w_{t}\right)$$

where *T* represents the length of the text sequence, *c* is the context window size, and $w_{t}$ is the central word. The skip-gram model predicts the surrounding contextual words given a central word.

For the skip-gram model, the input to the model is a one-hot vector of length *V*, representing the central word. This input vector is multiplied by a weight matrix *W* of size V×N (central word matrix), producing an *N*-dimensional hidden layer representation. The hidden layer output is then multiplied by another weight matrix $W^{\prime }$ of size N×V (context matrix) to generate a *V*-dimensional vector.

After applying the softmax function, the model outputs a probability distribution over the vocabulary. The word corresponding to the highest probability is treated as the predicted contextual word. If the predicted word does not match the actual contextual words, backpropagation is applied to update the weight matrices *W* and $W^{\prime }$.

#### 2.3.2. GcForest Building

We adopt **gcForest** [26] as the core model for clone detection, customizing it for our AST-Word2Vec representations. Our focus is on two key modules: **multi-grained scanning** and **cascade forest**, tailored to structured code data.

**(1)**  **Multi-Grained Scanning**

The multi-grained scanning module is designed to capture a range of feature representations for classification. It applies sliding windows over the raw feature vector to create sub-samples, which are then used for training both completely-random forests and traditional random forests. Each forest outputs a probability vector, and all vectors are concatenated to form the final feature representation.

We apply multi-grained scanning over AST-Word2Vec sequences to capture patterns at different granularities. Sliding windows of sizes 100, 50, and 25 are used to generate sub-samples from feature vectors of size p×d, where *p* is the number of AST nodes and *d* is the dimension of each node vector. The number of sub-samples, *S*, is computed as:

(4) $$S=\frac{p-w}{1}+1$$

Each sub-sample is processed by both completely-random forests and standard random forests to produce probability vectors. All vectors are concatenated to form a rich feature representation for each layer, enabling the model to capture both local and global structural patterns.

**(2)**  **Cascade Forest**

**Figure 4:**
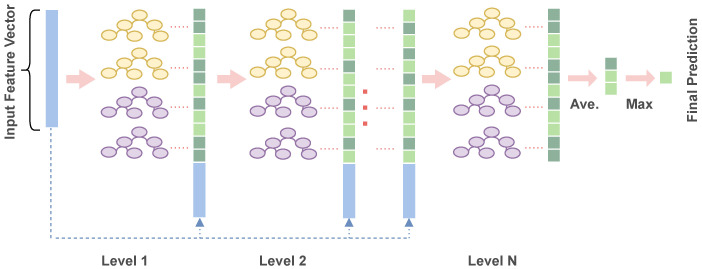
Overview of the Cascade Forest.

The cascade forest module processes feature vectors layer by layer, enhancing representational learning and improving classification accuracy. At each level, the input features are augmented with outputs from the previous layer. This design is particularly effective for sparse AST features, as it allows the model to gradually refine representations and enhance discriminative power. The overall architecture of the cascade forest module is illustrated in Figure 4.

Each layer employs multiple forest models (two completely-random forests and two standard random forests), and class vectors are computed using *K*-fold cross-validation to prevent test bias. The cascade continues until the last layer, where the final prediction is obtained by averaging class vectors and selecting the class with the highest value. The depth of the cascade forest is determined automatically during training.

GcForest is particularly well-suited for structured and sparse inputs such as AST-Word2Vec sequences. Compared with conventional deep neural networks, it robustly handles high-dimensional features with relatively few hyperparameter adjustments. Its cascade structure and ensemble design improve the detection of complex clones while maintaining stability on small or unevenly distributed datasets.

### 2.4. Recommendation Presentation

In the last step, RioCC post-processes the most relevant code files from the preceding stages, including the following two steps.

**Similarity Calculation.** The final code recommendation is determined by the similarity between the target code file and a candidate file, which is composed of two factors: quick search-based similarity (*SQscore*) and code clone-based similarity (*SCscore*). Thus, the final similarity can be calculated by a ranking function as the following equation:

(5) CScore=α·SQscore+β·SCscore

where *SQscore* and *SCscore* are generated by the stage of quick search (Section 2.2) and code clone detection (Section 2.3), respectively. Here, α and β are treated as fixed balancing coefficients used to combine the two complementary similarity components. They are kept constant across all experiments to ensure interpretability and fair comparison, rather than being tuned as sensitive hyperparameters.

In the recommendation stage, RioCC recommends the relevant codes mainly depending on the results of code clone detection, and the similarity of quick search (SQscore) will be considered when the similarity of code clone detection (SCscore) of different code pairs are equal (that is, the same code clone types). To achieve this goal, we adopt the similarity calculation strategy by assigning weight parameters α and β to the similarity SQscore and SCscore in Equation (5), respectively.

Apart from that, we calculate the quick search-based similarity between the target code and all code files, and then select the 1000 candidate codes ($N_{\#}$ = 1000) with the highest ranking similarity from the code pool. Specifically, we normalize these 1000 similarities into numbers between 0 and 1, following the equation of $s^{\prime }=\left(s-min\right)/\left(max-min\right)$, where *min* is the minimum similarity and *max* is the maximum one.

In the stage of code clone detection, we build the gcForest model to obtain the clone classification results of code pairs. However, since the output of this stage is a classification result of the clone type, we cannot obtain the similarity value directly. Therefore, we define each of the four clone types with a similarity score, referred to as code clone-based similarity. Specifically, the four types are assigned values of 0.125, 0.375, 0.625, and 0.875, respectively. This scoring scheme is designed to ensure that: (1) the scores are uniformly distributed within the range (0, 1) while avoiding extreme values, (2) the minimum gap of 0.25 between adjacent types maintains a clear separation in similarity levels, and (3) the values align with the commonly accepted intuition that a larger score indicates a higher similarity between the target code and the candidate code.

Previous work has shown that similarity threshold selection is highly sensitive to dataset characteristics, often requiring per-dataset tuning to achieve optimal performance [27]. Furthermore, recent systematic reviews highlight a lack of empirical evaluations for alternative scoring schemes [28,29]. In this context, our approach of evenly distributing scores with fixed gaps is both a pragmatic design choice intended to promote consistent behavior across datasets, and a response to this gap in the literature. Additionally, similarity-based metrics have been successfully used in software defect prediction tasks [30], indicating their practical utility. Since the minimum similarity difference between clone types is 0.25, the value range of α in Equation (5) is correspondingly defined as the interval (0, 0.25). To ensure that the final similarity value (CScore) falls within [0, 1], the α is set to 0.125 and β is set to 1 in practice. Alternative strategies, such as directly using the probabilistic outputs of gcForest or learning task-specific similarity functions, represent viable extensions to the current heuristic mapping. Similarly, more advanced rank aggregation methods could be employed to integrate heterogeneous similarity signals.

**Ranking Strategy.** After obtaining the similarity between the target code and all candidate code files, we straightforwardly recommend the top-*N* code files by sorting the similarity. To ensure the diversity of the recommending code files, the parameter *N* is set to 10. Thus, given a target code fragment, RioCC selects the first 10 code files and recommends them to the developer.

## 3. Experimental Setup

### 3.1. Research Questions

Since RioCC utilizes deep code clone detection techniques, we first evaluate the performance of deep forest in code cloning. Additionally, as RioCC’s primary goal is to recommend relevant code fragments to developers, it is essential to demonstrate its recommendation performance in real-world scenarios. Lastly, we also assess the efficiency of RioCC in terms of time consumption for code recommendation. Based on these objectives, we design empirical experiments to address the following research questions:

- **RQ1:** How does RioCC perform in detecting clone pairs compared to state-of-the-art methods?
- **RQ2:** How well does RioCC recommend real-world code fragments?
- **RQ3:** What is the time consumption of RioCC for code recommendation?

### 3.2. Subjects

The subjects used in this study are divided into two categories: datasets for code clone detection and datasets for the code pool, described below.

#### 3.2.1. Dataset for Code Clone

For training the gcForest model, we use the BigCloneBench dataset [31], which is a benchmark containing over 25,000 Java projects (365 million lines of code). It includes 10 folders with functions from different projects, 6 million labeled true clone pairs, and 260,000 labeled false clone pairs. These clone pairs cover all four clone types (T1, T2, ST3, and MT3/WT3/4), which correspond to the standard clone categories (Type-1, Type-2, Type-3, and Type-4) commonly used in clone detection research. We further divide them into four categories for performance comparison:

- NT1: T1 and T2 clones.
- NT2: VST3 and ST3 clones.
- NT3: MT3 and WT3/4 clones.
- NT4: Non-clone pairs.

To avoid noise from very short methods, we exclude methods with fewer than six lines of code. Since the number of T1-ST3 clone pairs is limited, we select 80% for training and 20% for testing. For MT3 and WT3/4 clones, we randomly select 40,000 pairs and similarly split them into training and testing sets. Additionally, we randomly select 80,000 non-clone pairs (NT4) for balanced training and testing. This results in a total of 192,000 clone pairs. The dataset distribution is shown in Table 1.

#### 3.2.2. Dataset for Code Pool

To recommend relevant code fragments, we built a code pool consisting of 106 open-source projects hosted on GitHub (https://github.com), each with more than 1000 stars. These projects span a variety of application domains, including music, video, text, and image processing. We excluded forked projects to ensure code quality, resulting in 396,277 class files.

Since the code pool lacks labeled data, we manually marked 400 random clone pairs as ground truth. All selected projects are Java-based, ensuring language consistency in the code pool. To ensure a balanced representation of each clone type (Type-1 to Type-4), we employed a stratified sampling strategy that guaranteed equal numbers of pairs per clone category. The labeling process was conducted manually by two experienced annotators with backgrounds in software engineering. Each annotator independently labeled the clone type of each code pair according to the established definitions of clone categories. Any disagreements were resolved through discussion and consensus. This rigorous annotation procedure ensured high-quality ground truth labels for subsequent evaluation. The resulting dataset was then used to assess the recommendation performance of RioCC, which combines both quick search-based and code clone-based similarity calculations, as described in Section 2.

### 3.3. Metrics and Baseline

#### 3.3.1. Metrics

To evaluate the performance of RioCC, we use three common metrics: *recall*, *precision*, and *F1-score*. Since code clone detection is a multi-class classification task, we calculate these metrics for each class separately.

Let $v_{ij}$ represent the number of instances where the true class is *j* and the predicted class is *i*. Given that we categorize the dataset into four classes, the recall, precision, and F1-score for class *k* are calculated as follows:

(6) $$Recall_{k}=\frac{v_{kk}}{\sum_{i=1}^{4}v_{ik}}$$

(7) $$Precision_{k}=\frac{v_{kk}}{\sum_{j=1}^{4}v_{kj}}$$

(8) $$F1\text{-}score_{k}=\frac{2\cdot Precision_{k}\cdot Recall_{k}}{Precision_{k}+Recall_{k}}$$

#### 3.3.2. Baseline

To evaluate the performance of RioCC, we consider several code clone detection tools as baselines, including RSharer [10], CCLearner [18], Oreo [19], SourceCC [9], Nicard [32], and Decard [33]. Among these, CCLearner, Oreo, and RSharer are based on deep learning techniques, which share similar data representation and learning paradigms with RioCC, allowing for a more direct comparison. SourceCC, Nicard, and Decard, on the other hand, utilize traditional tree-based or graph-based approaches, which differ fundamentally in methodology and feature extraction. Due to these inherent differences, directly comparing these methods with RioCC may not accurately reflect their relative strengths. Therefore, we focus on comparing RioCC with deep learning–based methods in this study.

- CCLearner: Extracts tokens from source code clones to train a DNN model for classification.
- Oreo: Employs a Siamese neural network to train the clone detection model.
- RSharer: Uses a CNN for the classification task.

For a fair comparison, we extend these three methods to handle multi-class classification tasks, as done with RioCC. It should be noted that the baselines listed above are primarily used for clone detection evaluation (RQ1). Due to differences in task objectives and output formats, only CCLearner is adopted as a baseline for recommendation quality evaluation in RQ2.

### 3.4. Experimental Setting

The experiments are conducted on an Intel(R) Xeon(R) CPU E5-2578 v3 2.5 GHz with 64 GB of memory and a GNU/Linux OS.

For preprocessing, we use the Eclipse ASTView plugin to extract AST structures from the code fragments. For Word2Vec training, we set the negative sampling size to 10, the embedding dimension to 64, the window size to 5, and the minimum word frequency to 3 to optimize training time. These hyperparameter values were determined empirically through preliminary experiments to balance training efficiency and embedding quality on our dataset.

Inspired by the previous work [26], for gcForest training in RioCC, we configure the multi-grained scanning module with sliding window sizes of 100, 50, and 25. The number of random forests is set to 2 (one completely-random tree forest and one random forest), each containing 500 decision trees. In the cascade forest module, the number of random forests per layer is set to 4 (two completely-random tree forests and two random forests), each with 1000 decision trees. Decision trees continue to grow until each leaf node contains only instances of the same class or no more than 10 instances. These parameters were further fine-tuned on a validation set to optimize classification performance for our dataset.

## 4. Experimental Results

In this section, we present the experimental results to answer the research questions posed in Section 3.1.

### 4.1. Performance of RioCC in Clone Pair Detection (RQ1)

#### 4.1.1. Clone Detection on BigCloneBench (RQ1-1)

Table 2 presents the clone detection results for various approaches: CCLearner, Oreo, RSharer, and RioCC. The clone detection task is treated as a four-class classification problem, with results shown in a 4 × 4 matrix for each method.

From the results, we observe that RioCC outperforms all other methods across all clone types, particularly in detecting NT3 and NT4, with correct detections of 11,921 and 12,264 pairs, respectively. Overall, the word embedding-based approaches (RSharer and RioCC) outperform the hand-crafted feature-based approaches (CCLearner and Oreo).

RSharer and RioCC leverage CNN and gcForest, respectively, to extract semantic and structural information, which enhances detection accuracy. The use of Word2Vec for preprocessing code further improves classification performance. Additionally, both methods employ sliding windows for sub-sampling, capturing more potential features. In contrast, CCLearner and Oreo rely on hand-crafted features, which limits their ability to learn complex patterns.

RioCC surpasses RSharer in performance, primarily due to its multi-grained scanning module within gcForest, which extracts fine-grained contextual information and enhances the model’s representational power.

Among the two traditional deep learning models, Oreo outperforms CCLearner on all datasets. Oreo benefits from more effective feature extraction, such as semantic signatures and software metrics, and from its Siamese architecture, which handles symmetry in input vectors and ensures accurate similarity measurements.

**Figure 5:**
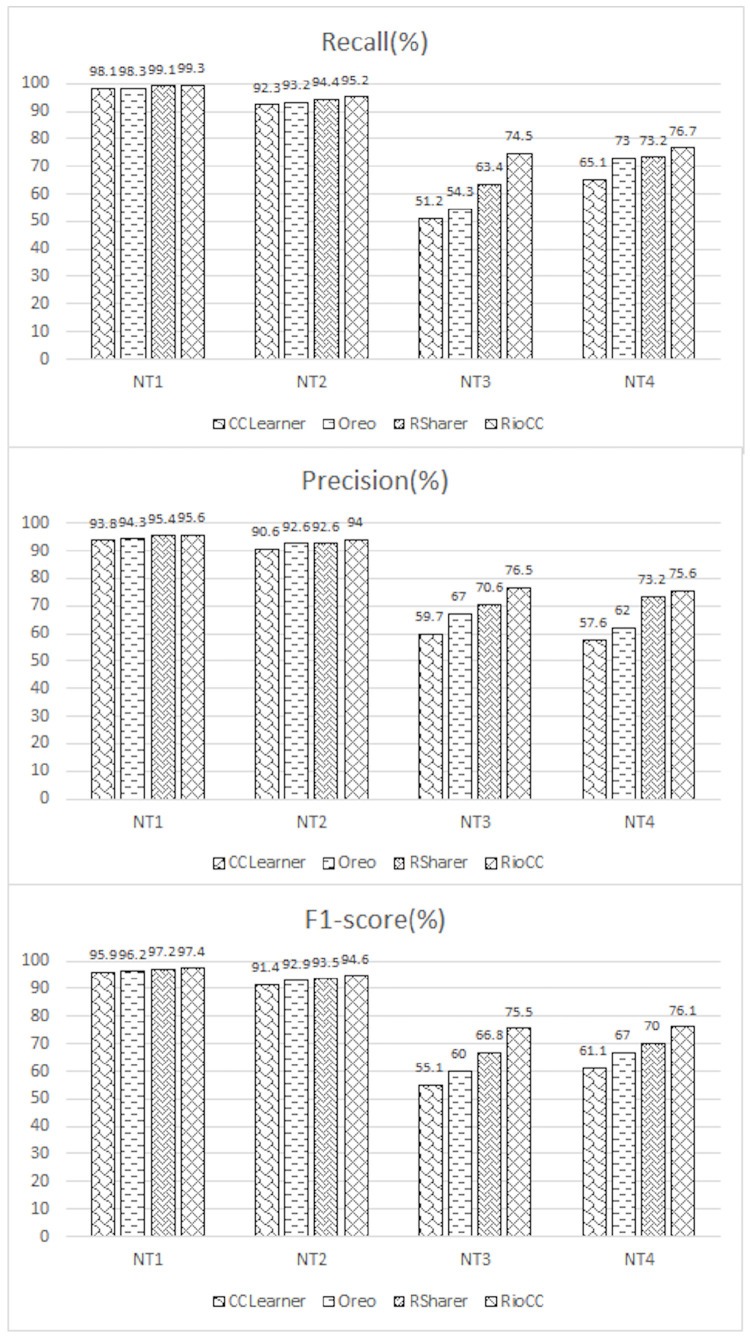
Comparison Results of Clone Detection for BigCloneBench.

Figure 5 shows the precision, recall, and F1-score for each approach on BigCloneBench. RioCC consistently achieves superior performance across all metrics, with F1-scores of 97.4% for NT1 and 94.6% for NT2. RSharer performs comparably in detection but does not match RioCC in fine-grained accuracy.

In general, while RioCC shows a clear advantage in detecting NT3 and NT4, it is not surprising that there is little difference in performance for NT1 and NT2, as these are relatively easier to detect for all approaches.

#### 4.1.2. Clone Detection on the Code Pool (RQ1-2)

Table 3 presents the clone detection results for the 400 manually marked code pairs in the code pool. RioCC clearly outperforms all other approaches across the four clone types, achieving the highest detection accuracy.

**Figure 6:**
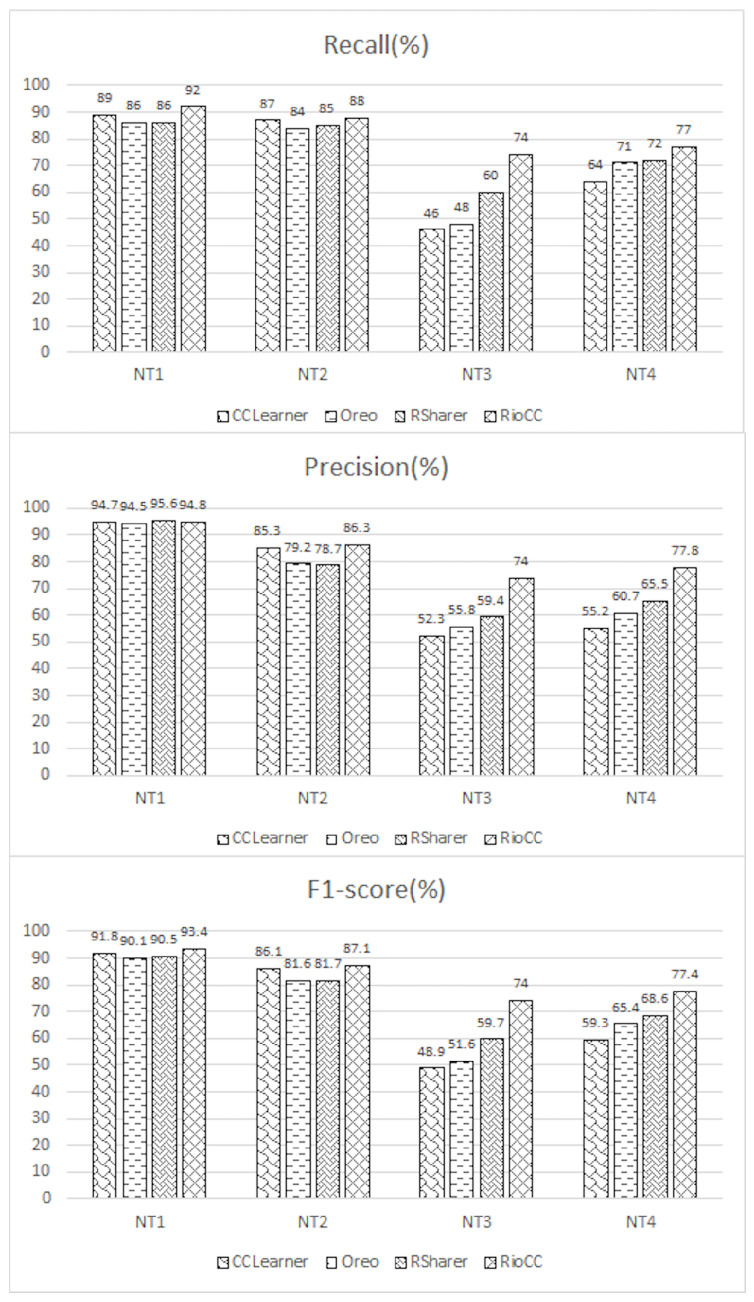
Comparison Results of Clone Detection for Code Pool.

Figure 6 compares the precision, recall, and F1-score of the different methods. RioCC shows a significant performance boost, with F1-score improvements of 14.3% for NT3 and 8.8% for NT4. Compared to BigCloneBench, RioCC maintains consistent performance on the code pool, with only slight variations in the F1-score for NT3 and NT4 between the two datasets. Interestingly, CCLearner outperforms Oreo and RSharer in detecting NT1 and NT2 on the code pool dataset, highlighting the sensitivity of traditional models to dataset characteristics.

Overall, these results reinforce the advantages of RioCC in both code representation and clone pair detection, demonstrating its robustness across different datasets.

### 4.2. The Performance of RioCC in Recommending Real-World Code Fragments (RQ2)

To ensure consistency and objectivity, we randomly selected 200 real code fragments and manually assessed the top-10 recommended code fragments for each. The manual evaluation was performed by two experienced software engineers who independently judged whether each recommended fragment belonged to one of the clone types relevant to the target code, based on the clone type definitions used in this study. To ensure consistency and objectivity, any disagreements were resolved through discussion until consensus was reached. As is common in large-scale recommendation systems, lower-ranked results are more likely to contain weakly related recommendations. Accordingly, we include a qualitative example from the lower end of the top-10 results to illustrate a representative error case.

As described in Section 2.4, RioCC recommends the top-10 fragments to programmers, and we analyzed the number of clone types among these recommendations. CCLearner was used as the baseline model for comparison. CCLearner was selected as the baseline model because it is a representative deep learning clone detector and provides outputs that can be readily aggregated at the class level. We emphasize that this case study is illustrative rather than exhaustive, and other methods are not included in the comparison.

Table 4 shows the average number of clone types in the top-10 recommendations for both RioCC and CCLearner. Ideally, we want recommendations to include code fragments related to the target code (NT1, NT2, and NT3) and avoid irrelevant code (NT4). The results reveal that, on average, RioCC recommends 3.1 NT2 and 1.8 NT3 code fragments, outperforming CCLearner, which recommends fewer NT2 and NT3 fragments. Additionally, CCLearner recommended an average of 1.8 NT4 code fragments, while RioCC only recommended 1.1, indicating that RioCC offers more relevant recommendations.

**Figure 7:**
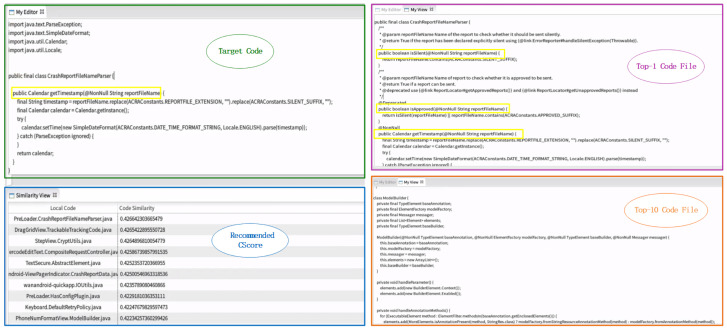
Recommendation Results of RioCC.

To provide a clearer understanding, we present a typical code sharing example. The target code fragment parses a crash report file for “Calendar,” extracting and setting the timestamp. Figure 7 illustrates the recommendation system in action. On the left, the input code and recommendation list are shown, with the right side displaying the top-1 and top-10 recommended code fragments.

The top-1 recommendation includes two additional methods to assess the crash report file, which could provide valuable context to developers. In contrast, the 10th recommendation contains a class for model building unrelated to the task at hand, demonstrating that lower-ranked recommendations are less relevant to the target code. These results highlight RioCC’s effectiveness in providing accurate and contextually relevant code recommendations.

### 4.3. Time Consumption of RioCC for Code Recommendation (RQ3)

While RQ1 and RQ2 focused on the accuracy and quality of recommendations, RQ3 isolates the efficiency aspect, measuring the time required to generate the recommendation. To evaluate the efficiency of RioCC, we conducted two comparative experiments using 200 randomly selected code fragments (as described in Section 4.2). The task was to generate the top-1 recommendation for each fragment and calculate the average recommendation time across all models.

In the first experiment, we tested the quick search-based RioCC and other models on the marked code pool, which contains over 300,000 code fragments, resulting in over six million possible clone pairs. In this scenario, all models took more than one day to recommend a single code fragment, which is impractical for real-world applications. In contrast, RioCC’s quick search module completed the recommendation in just 1.13 min on average.

**Figure 8:**
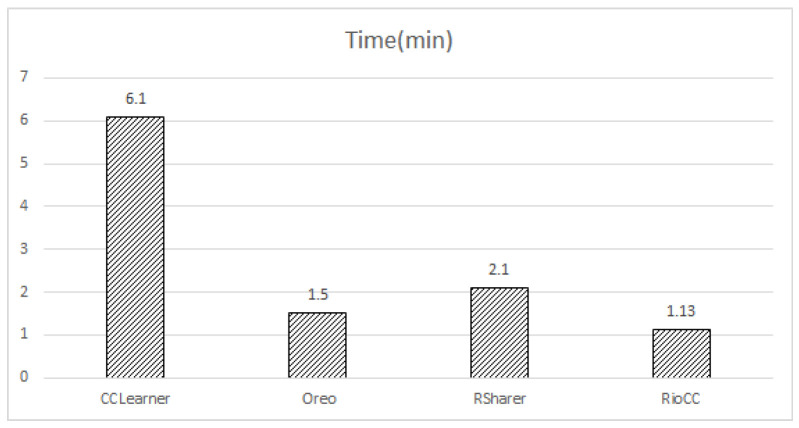
Comparison of Time Consumption.

In the second experiment, we aimed for a fairer comparison by pre-processing all models with the quick search module before generating recommendations. As shown in Figure 8, RioCC still achieved the lowest average time consumption compared to the other models. Oreo, which uses a size-based heuristic algorithm, was the second fastest, taking 0.37 min longer than RioCC. CCLearner, on the other hand, took the longest time, likely due to its two token extraction methods (ANTLR and Eclipse ASTParser) and its fully connected DNN model.

Overall, all models saw significant time reductions after applying the quick search, further validating its effectiveness in narrowing down the candidate code fragments for recommendation.

## 5. Limitations and Validity Concerns

### 5.1. Limited Dataset

We trained our clone detection model on BigCloneBench, which, despite containing a large number of true clone pairs, is restricted to ten function types. Additionally, our code pool consists of selected GitHub projects, which may not fully represent diverse real-world scenarios. This dataset limitation could impact the stability of RioCC’s recommendations, potentially leading to biased results in real deployments. Furthermore, both BigCloneBench and our code pool contain only Java-based code fragments, which limits the generalizability of our approach to other programming languages. Expanding the dataset with more real-world clone pairs and diverse programming languages is part of our future work.

### 5.2. Manual Evaluation Bias

Code recommendation performance was evaluated through manually labeled code pairs, which may introduce bias due to differences in domain knowledge among evaluators. To mitigate this, each code segment was reviewed by at least two authors, and the final evaluation was determined by averaging their assessments. However, no formal inter-annotator agreement metric (e.g., Cohen’s κ) was computed, which represents a potential threat to evaluation validity. Future work will incorporate formal inter-annotator agreement analysis to further strengthen the robustness of the evaluation. Furthermore, we note that a detailed error analysis or misclassification breakdown across clone types was not conducted due to the lack of preserved prediction logs. Such analysis could provide deeper insights into failure cases and is left for future work. Accordingly, the qualitative example discussed in Section 4.2 is intended to illustrate a typical error case rather than to provide a comprehensive error analysis. In addition, the current study focuses exclusively on Java code, and the evaluation is conducted on Java-based datasets. While the overall framework of RioCC is general, the feature extraction and program representations are language-dependent; therefore, whether similar performance trends would hold for other programming languages remains an open question and is left for future work.

### 5.3. Limited Availability

Currently, RioCC is implemented as an Eclipse plugin, which restricts its use primarily to Java developers. Exploring broader implementation formats is an important direction for enhancing the accessibility of the approach. Moreover, RioCC recommends class-level code fragments based solely on similarity, without explicitly considering factors such as developer intent or coding style. Incorporating such contextual information could further improve recommendation quality in real-world applications. Finally, the current heuristic scheme could be extended by integrating probabilistic outputs from gcForest, learned similarity functions, or rank aggregation strategies to better combine heterogeneous similarity signals.

## 6. Discussion

This section provides a qualitative discussion of the differences between RioCC and recent LLM-based approaches for code recommendation. We do not aim to present a numerical or experimental comparison, as these methods are designed for different problem settings and evaluation paradigms.

- **Advantages of RioCC:** RioCC excels in program recommendation by leveraging structured feature extraction, multi-grained scanning, and gcForest-based classification. Unlike deep learning models that require extensive labeled data, RioCC benefits from an ensemble-based learning approach, reducing dependence on large-scale pretraining. Additionally, the quick search module significantly improves efficiency by narrowing down the search space, making RioCC highly scalable for large codebases. These characteristics ensure that RioCC provides precise and computationally efficient code recommendations, particularly for structured clone detection tasks.
- **Advantages of LLM-based Approaches:** Recent advancements in large language models (LLMs), such as GPT [34] and CodeBERT [21], have revolutionized program recommendation by capturing deep contextual and semantic relationships in source code [35,36,37]. LLMs can generate meaningful recommendations even for unseen code structures, generalizing well across different programming paradigms. Their ability to learn from vast corpora enables them to recommend code fragments that align with developers’ intent, making them particularly effective in open-ended, generative tasks like code synthesis, refactoring suggestions, and intent-driven search.
- **Scenarios Where RioCC Remains Advantageous:** Despite the strengths of LLMs, RioCC remains highly effective in specific scenarios. In structured clone detection tasks where precise similarity measurement is crucial, RioCC offers deterministic and explainable results, which LLMs may struggle with due to their probabilistic nature. Additionally, for domains requiring strict control over training data and interpretability—such as safety-critical software or enterprise applications—RioCC’s structured, feature-driven approach remains preferable. Moreover, RioCC is computationally lightweight compared to LLMs, making it more suitable for real-time recommendation tasks with limited computing resources.

In conclusion, while LLMs provide a promising alternative for program recommendation, RioCC remains a strong choice for structured clone detection, efficient large-scale code retrieval, and scenarios demanding high interpretability and computational efficiency. Future work could explore integrating LLM-based techniques with RioCC to combine their strengths, further enhancing code recommendation performance.

## 7. Related Work

### 7.1. Code Recommendation

Code recommendation helps developers write efficient code and detect bugs. Existing methods include code-to-code search tools [2,3,4] and pattern-based code completion techniques [5,6,7]. For the first type, Kim et al. [3] introduced FaCoY, a search tool that recommends semantically similar code snippets by leveraging code descriptions from Stack Overflow. Given an input code snippet, FaCoY identifies relevant code examples through semantic matching, providing developers with meaningful references. Luan et al. [2] proposed Aroma, a structural code search tool that generates comprehensive recommendations by combining multiple similar-looking code snippets rather than suggesting a single method body. This approach effectively broadens the scope of code recommendations by presenting diverse yet related code examples. Unlike code-to-code search methods, pattern-based code completion approaches recommend code snippets by matching a set of pre-mined patterns, such as API usage patterns. These methods focus on improving keyword-based queries to retrieve relevant code examples and API documentation for developers. While clone detection techniques have been explored for code recommendation, they primarily identify highly similar code fragments, limiting their ability to suggest meaningful code extensions.

To overcome this, we propose a class-level code recommendation tool that combines clone detection with the gcForest technique. By capturing both syntactic and semantic structures, our method extends partially written code snippets, offering developers more comprehensive code references and bridging the gap between clone detection and effective code recommendation.

### 7.2. Code Clone Detection

Code clone detection techniques can be broadly categorized into four types: text-based [38,39,40], token-based [9,41], tree-based [18,33], and graph-based [42,43,44,45]. Text-based techniques are simple and efficient but struggle with detecting complex clones, particularly those involving variable contexts [46]. Token-based techniques analyze token sequences [28], offering improved robustness against code variations and excelling in identifying Type-2 clones. Tree-based techniques map code fragments to AST or similar structures and apply tree-matching algorithms to compare them [47]. This method effectively detects near-miss clones by capturing syntactic similarities.

Graph-based techniques convert code fragments into graph representations such as program dependence graphs (PDG) [9] and control flow graphs (CFG) [48]. These techniques leverage high-level abstractions and rich semantic information, making them suitable for identifying near-miss clones. Despite their strengths, existing techniques face challenges in balancing detection accuracy with computational efficiency. Graph-based methods, in particular, often incur high time and memory costs [9]. Furthermore, accurately identifying semantic-aware clones (Type-3 and Type-4) remains difficult. Unlike conventional tree-based methods, our approach employs a tree ensemble model to effectively capture both syntactic and semantic structures. This design improves the detection of complex code clones while maintaining computational efficiency.

In addition to classifications based on representation methods, code clone detection research can also be categorized according to the granularity of code analyzed. Existing studies focus on various levels: method- or function-level clones that detect similar code fragments within individual functions or methods [21,22]; class-level clones considering entire classes as the basic unit for similarity detection [23]; and system-level clones comparing larger software components or entire systems [24]. Our method is specifically targeted at class-level clone detection, aligning it with this established category in clone detection research.

### 7.3. LLM-Based Code Engineering

Recently, LLMs have demonstrated remarkable capabilities across various domains, including natural language processing, computer vision, and speech recognition [49]. With extensive code-related tasks included in their pre-training data, LLMs have become increasingly popular in addressing software engineering challenges. Additionally, specialized LLMs tailored for code-related tasks have emerged [50,51], finding wide applications in code generation, repair, and optimization.

Feng et al. [52] proposed an automated method that leverages prompt engineering to reproduce bugs from bug reports. Deng et al. [53] introduced a testing framework that employs generative and infilling LLMs to create and modify diverse programs for evaluating deep learning libraries. Han et al. [54] utilized Claude-3-haiku for its strong semantic understanding and efficient processing of large-scale codebases, while Nichols et al. [55] adopted Gemini-Pro-1.0 for generating synthetic code snippets, demonstrating superior performance in their experiments.

LLMs have also been explored for improving code clone detection. Dou et al. [56] conducted a comprehensive evaluation of various LLMs, including LLaMA [57,58], Alpaca [59], and GPT [60], demonstrating that advanced LLMs excel at identifying complex semantic clones. Gong et al. [61] further evaluated 61 general-purpose LLMs in code optimization and related tasks, encompassing models from the GPT family [62,63], LLaMA family [64,65,66], Claude family [54,62], and other open-source models [67,68]. While their findings highlight LLMs’ impressive potential, they also reveal notable limitations. The increasing complexity and resource demands of modern LLMs pose challenges for practical deployment. Moreover, most LLM-based code optimization methods operate in isolated environments, lacking the dynamic interaction with external systems that human programmers routinely rely on—such as internet searches, external tools, and peer collaboration—to achieve superior code improvements.

## 8. Conclusions

In this paper, we introduced RioCC, a novel code recommendation tool designed to assist developers in identifying functionally similar yet correctly implemented code snippets from related projects. Our key contributions include: (a) proposing a class-level recommendation approach to provide richer contextual information, (b) leveraging the advanced deep forest technique to effectively learn code clones from BigCloneBench, and (c) integrating a quick search module with a filtering strategy to discard irrelevant candidates, significantly improving search efficiency. Our quantitative evaluation demonstrates that RioCC outperforms state-of-the-art clone detection methods, including CCLearner, Oreo, and RSharer, in both recommendation accuracy and efficiency when applied to real-world code fragments. For future work, we plan to expand RioCC by exploring alternative implementations and evaluating its effectiveness on broader real-world datasets.

## Figures and Tables

**Table 1:** BigCloneBench Dataset.

Dataset	NT1		NT2		NT3		NT4
	T1	T2	VST3	ST3	MT3	WT3/4	Non-Clone Pairs
Training	12,800	2880	1600	8000	32,000	32,000	64,000
Testing	3200	720	400	2000	8000	8000	16,000

**Table 2:** Clone Detection Results in BigCloneBench. NT1/2/3/4^*D*^ is the number of detected clone pairs. NT1/2/3/4^*T*^ is the number of true clone pairs. The bold numbers refer to the number of clone pairs that are correctly classified.

Method		NT1^*D*^	NT2^*D*^	NT3^*D*^	NT4^*D*^	Total
CCLearner	${\mathrm{NT}1}^{T}$	**3845**	102	87	63	4097
	${\mathrm{NT}2}^{T}$	42	**2215**	97	91	2445
	${\mathrm{NT}3}^{T}$	20	67	**8192**	5434	13,713
	${\mathrm{NT}4}^{T}$	13	16	7624	**10,412**	18,065
Oreo	${\mathrm{NT}1}^{T}$	**3853**	98	82	54	4087
	${\mathrm{NT}2}^{T}$	39	**2236**	84	56	2415
	${\mathrm{NT}3}^{T}$	23	48	**8688**	4217	12,976
	${\mathrm{NT}4}^{T}$	16	18	7146	**11,673**	18,853
RSharer	${\mathrm{NT}1}^{T}$	**3885**	71	64	53	4073
	${\mathrm{NT}2}^{T}$	24	**2265**	81	77	2447
	${\mathrm{NT}3}^{T}$	11	42	**10,144**	4167	14,364
	${\mathrm{NT}4}^{T}$	0	22	5711	**11,708**	17,441
RioCC	${\mathrm{NT}1}^{T}$	**3892**	67	59	53	4071
	${\mathrm{NT}2}^{T}$	17	**2285**	68	62	2432
	${\mathrm{NT}3}^{T}$	11	33	**11,921**	3621	15,586
	${\mathrm{NT}4}^{T}$	0	15	3952	**12,264**	16,231
Total		3920	2400	16,000	16,000	38,320

**Table 3:** Clone Detection Results in Code Pool. NT1/2/3/4^*D*^ is the number of detected clone pairs. NT1/2/3/4^*T*^ is the number of true clone pairs. The bold numbers refer to the number of clone pairs that are correctly classified.

Method		NT1^*D*^	NT2^*D*^	NT3^*D*^	NT4^*D*^	Total
CCLearner	$\mathrm{NT}1^{T}$	**89**	3	2	0	94
	$\mathrm{NT}2^{T}$	7	**87**	4	4	102
	$\mathrm{NT}3^{T}$	4	6	**46**	32	88
	$\mathrm{NT}4^{T}$	0	4	48	**64**	116
Oreo	$\mathrm{NT}1^{T}$	**86**	4	1	0	91
	$\mathrm{NT}2^{T}$	10	**84**	11	1	106
	$\mathrm{NT}3^{T}$	3	7	**48**	28	86
	$\mathrm{NT}4^{T}$	1	5	40	**71**	117
RSharer	$\mathrm{NT}1^{T}$	**86**	2	2	0	90
	$\mathrm{NT}2^{T}$	8	**85**	12	3	108
	$\mathrm{NT}3^{T}$	5	11	**60**	25	101
	$\mathrm{NT}4^{T}$	0	2	36	**72**	110
RioCC	$\mathrm{NT}1^{T}$	**92**	4	1	0	97
	$\mathrm{NT}2^{T}$	7	**88**	5	2	102
	$\mathrm{NT}3^{T}$	1	6	**74**	19	100
	$\mathrm{NT}4^{T}$	0	2	20	**77**	99
Total		100	100	100	100	400

**Table 4:** The number of different clone types for 10 recommended results.

Model	NT1	NT2	NT3	NT4
RioCC	4	3.1	1.8	1.1
CCLearner	4	3	1.2	1.8

## Data Availability

The data used in this study were obtained from publicly available sources and have been cited in the manuscript.
